# Who Gets to Breastfeed? A Narrative Ecological Analysis of Women's Infant Feeding Experiences in the UK

**DOI:** 10.3389/fsoc.2022.904773

**Published:** 2022-07-22

**Authors:** Gill Thomson, Jenny Ingram, Joanne Clarke, Debbie Johnson, Kate Jolly

**Affiliations:** ^1^School of Community Health & Midwifery, University of Central Lancashire, Preston, United Kingdom; ^2^Centre for Academic Child Health, Bristol Medical School, University of Bristol, Bristol, United Kingdom; ^3^Institute of Applied Health Research, University of Birmingham, Birmingham, United Kingdom

**Keywords:** ecological, breastfeeding, women, interviews, systems level, qualitative, infant feeding

## Abstract

The early post-natal period is a critical period in women's infant feeding journeys, often marked by high levels of unintended breastfeeding cessation. Previous research has argued that infant feeding should be perceived within a complex system whereby factors operating at different ecological levels (i.e., individual, social/community networks, cultural/institutional) interact to affect individual behaviors. However, currently, more work needs to be done to implement an ecological approach in breastfeeding programs. We adopted a complex-systems lens approach to explore how multi-level factors—individual, mother-infant dyad, health service, family and social networks, and wider community infrastructure—interacted with women's motivations and experiences of breastfeeding. We undertook a secondary analysis of 24 women's interviews; all the women had a strong antenatal intention to breastfeed and were expecting their first baby. The interviews were collected during the UK-based Assets-based feeding help Before and After birth (ABA) feasibility trial when their infant was aged between 4 and 21 weeks. Categorical content analysis was used to explore the interrelationships between key factors and to identify different infant feeding typologies. Two different typologies emerged: “disappointed” (*n* = 7) and “by hook or by crook” (*n* = 17). “Disappointed” women had stopped breastfeeding early; women classified as “by hook or by crook” continued breastfeeding despite facing challenges. Sociodemographic, social, and service level differences between the typologies were noted. “Disappointed” women were more likely to be younger, White-British, to have considered mixed-feeding antenatally and experienced negative breastfeeding support from healthcare professionals and personal networks. Infants of “disappointed” women were more likely to have received unexpected “top-ups” and to be perceived as having infant feeding difficulties. Women classified as “by hook or by crook” were just as likely as “disappointed” women to experience birth-related complications, but demonstrated more proactive help-seeking behaviors, had positive experiences of personal/professional support and accessed wider support. While further research is needed to consolidate and/or refute the typologies, the ecological approach shifts the focus away from mothers' decisions to consider the multi-level factors that need to be in place to enable women to breastfeed successfully. Further work to encourage help-seeking behaviors and toward improving facilities, support, and services is needed.

## Introduction

In the UK, the early days and weeks post-birth have been identified as a critical period in women's infant feeding journeys, marked by very high levels of unintended breastfeeding cessation (McAndrew et al., 2012). When asked why they have stopped breastfeeding, women often describe feeding problems such as painful breasts or nipples, difficulties in attaching the baby to the breast, and perceived low milk supply (Li et al., 2008; McAndrew et al., 2012). These explanations for breastfeeding cessation, self-reported by individual mothers, understandably draw our attention to individual-level explanations for outcomes.

There is abundant evidence and arguments that infant feeding decisions need to be perceived within a complex system of influences that operate at different ecological levels (Dyson et al., 2006; Rollins et al., 2016; Trickey, 2017, 2018; Trickey et al., 2018; Tomori et al., 2022; Vilar-Compte et al., 2022a,b). A complex systems approach asserts that the “properties of the parts can only be understood within the larger context of the whole” (Merali and Allen, 2011, p. 32); it considers the importance of the agency of individuals within the system and the parameters of the system that individuals inhabit. These include the health service, family and social networks, and wider community infrastructure. These observations form part of a contextual turn in thinking about ways to address low breastfeeding rates (Rollins et al., 2016; UNICEF, 2018). This ecological approach shifts the focus away from mothers' decisions and toward improving facilities, support, and services at the community level and by addressing socio-economic, cultural, environmental, and legislative barriers at the national level (Trickey, 2017).

Maternal self-efficacy has been found to be key to infant feeding journeys. Mothers who feel confident about their ability to breastfeed successfully are more able to overcome barriers to breastfeeding (Entwistle et al., 2010). Interventions to improve self-efficacy, for example through antenatal education (McQueen et al., 2011; Galipeau et al., 2018) and through peer support (Ingram, 2013; Moudi et al., 2016) have indicated that women are more likely to breastfeed exclusively for longer (Brockway et al., 2017; Galipeau et al., 2018). However, little is known about the ways in which self-efficacy interacts with and is shaped by wider contextual factors. For example, research suggests that the women's family and social networks play a key role in shaping women's self-efficacy for breastfeeding (Brockway et al., 2017). Women who have been breastfed as an infant (Murimi et al., 2010), and know others who have breastfed (Thomson et al., 2012) are more likely to breastfeed. Furthermore, the service context for decisions, including the knowledge, beliefs, and attitudes of health professionals, appear to act as a controlling parameter in shaping the success of interventions that are intended to improve breastfeeding self-efficacy and breastfeeding rates (Brockway et al., 2017; Trickey, 2018). Indeed, it has been argued that interventions to improve maternal self-efficacy may struggle to gain purchase in a service pathway that is “broken,” so that their influence becomes primarily remedial (Trickey, 2018). While there is strong evidence of multi-component and multilevel interventions in other public health arena (e.g., Kothari et al., 2007; Lane et al., 2016; Tehrani et al., 2016), this is not the case within an infant feeding arena where most interventions target the health service only (Tomori et al., 2022). This may be due to a lack of understanding of complex systems thinking within an infant feeding agenda, where there is little known about the ways in which the service context interacts with a social network context and with maternal intention and motivation—in other words, the extent to which system parameters ultimately shape mothers' intentions and motivations to breastfeed (Trickey, 2017, 2018).

This paper reports on a secondary analysis of a sub-set of data collected as part of the Assets-based feeding help Before and After birth (ABA) feasibility trial which was conducted from November 2016 to October 2018 (Clarke et al., 2020a,b). We applied a complex systems lens to interview data that captured women's own accounts of feeding their babies through the early weeks to explore how wider contextual conditions interacted with women's decision-making. We aimed to understand how different influences within a system of influences—the mother-infant dyad, the health service, family and social networks, and wider community infrastructure—interacted with women's motivations and experiences of breastfeeding.

## Materials and Methods

### Setting

The ABA study was a feasibility randomized controlled trial for the ABA assets-based peer support intervention undertaken at two geographical sites in England (Clarke et al., 2020a,b). The sites were selected for having high levels of socioeconomic deprivation and low rates of breastfeeding initiation and continuation. The ABA study explored the feasibility, acceptability, and utility of a new model of peer support. The model was underpinned by behavior change theory and offered an assets-based approach that focussed on positive capabilities of individuals and communities, including use of a Genogram (aka friends and family diagram) and assets leaflet (Clarke et al., 2020a; Ingram et al., 2020; Thomson et al., 2020). Trained peer supporters (named Infant Feeding Helpers) provided women with an antenatal contact (~30–32 weeks gestation), daily support (text, telephone) for 2 weeks immediately after birth, and for ongoing support up to 8 weeks post-natal, with frequency and types of contact organized on a peer-mother negotiated basis (Clarke et al., 2020a,b).

### Recruitment

Eligible women were 16 years or above and pregnant with their first baby. Study information was provided by community midwives and women were then approached by a researcher in antenatal clinics to gain informed consent. In total, 103 women participated in the study. Women were randomized to two groups, one receiving usual care and the other having usual care (i.e., infant feeding support provided by midwives, health visitors and *via* wider community support that a woman may access, e.g., breastfeeding groups) plus the ABA intervention.

### Participants

In the weeks after the birth, thirty women (21 intervention and 9 control) from the two ABA study sites were interviewed in detail about their infant feeding experiences. These participants were purposively sampled to include younger mothers and women living in areas of socioeconomic disadvantage, and to include women with a range of baseline feeding intentions.

### Data Collection

As part of the ABA study, pregnant women completed a baseline questionnaire at recruitment; this included demographic questions and asked women how they were fed as a baby and if they know anyone who has breastfed their baby. Women were also asked to report their feeding intentions (using an open text question). In a follow up questionnaire completed at 8 weeks post-natal, women reported type of birth and feeding method.

Postnatal interview guides were semi-structured—women were prompted to talk in general about their experiences of feeding their baby, any difficulties they had encountered and any support they had received as well as about any advice or suggestions they would want to pass on to a friend based on their own experience (examples of interview questions are detailed in Table 1). Interviews took place in the woman's home between June 2017 and February 2018, lasted between 45 and 90 min and were voice-recorded, transcribed verbatim, checked for accuracy and anonymised. Each story captured insights into experiences of feeding and feeding support from the antenatal period up until the point of interview. Interviews with the first four participants took place when their babies were aged 4–6 weeks; interviews with the remaining participants (*n* = 26) took place when babies were aged 8–21 weeks. Overall, there was no notable differences in the content of interviews undertaken at different post-natal time points.

**Table 1:** Examples of interview questions.

The mother's feeding story	Can you tell me about your experience of feeding your baby …?
	Were there any challenges or difficult times in terms of feeding your baby?
Antenatal feeding help	Thinking back to before your baby was born … how were you thinking about feeding your baby?
	How different is your experience to what you had expected?
	Is there anything they would say to friends who are pregnant for the first time to help them prepare?
Postnatal feeding help	Can you tell me about your experience of infant feeding help in the hospital?
	Can you tell me about other types of help you have received for infant feeding—so any help you have received from health professionals, friends, family, other support?
	Can you tell me about any times when you particularly needed help with feeding your baby—what happened?
	Thinking about immediate family, friends, health professionals and anyone else who has been around … who do you feel has been most helpful to you with feeding your baby?

### Data Analysis

For the purposes of this study, we undertook a secondary analysis of a sub-set of women's interviews to explore the systems level factors that influenced women's motivations and experiences of breastfeeding. As part of the initial feasibility study (Clarke et al., 2020b), the free text information on feeding intention in the baseline questionnaire was categorized by two researchers using the scale developed by Hoddinott and Pill (1999; see Table 2). Any discrepancies in categorization were resolved through discussion.

**Table 2:** Classification of breastfeeding intentions (Hoddinott and Pill, 1999).

**Feeding intention**	**Description**
Committed breast feeder	Refers to perseverance, overcoming/coping with problems; don't mention anticipated problems
Probable breast feeder	Express “some” doubt about own and other women's abilities to breastfeed
Possible breast feeder	Less committed and mention scenario where they would change their feeding intention
Probable formula feeder	Initially say they will formula feed, but also that they “might” consider breastfeeding
Committed formula feeder	Do not mention considering breast feeding
Not classified	No indication of feeding intention provided

For the purposes of this study, we only focused on the narratives of women who had strong antenatal intentions (classified as strong or probable) to breastfeed (*n* = 24); women who were classified as *committed formula feeders* (*n* = 2), *possible breast feeders* (*n* = 2) or *unclassified* (*n* = 2) were excluded from the analysis. This was to enable an in-depth understanding of different factors that facilitate or impeded women's motivations to breastfeed, and to prevent biasing our analysis by including those who held equivocal or positive intentions to formula feed.

We then used a categorical-content analysis method to analyse and interpret the data set, based on the approach described by Iborra (2007) and Gondim and Bendassolli (2014). This approach enables narrative data to be processed analytically by breaking the text into relatively small units of content to examine thematic similarities and differences. As described by Lieblich et al. (1998) “*the narrative story is dissected, and sections or single words belonging to a defined category are collected from the entire story or from several texts belonging to a number of narrators*” (p. 12). We considered this approach most appropriate to enable us to identify and to understand all the multi-system level factors that influenced women's experiences of breastfeeding. Additionally, and to address criticisms about how this approach can create a detached analysis (Conrad, 1990), we also developed narrative case studies (in the form of typologies) to contextualize women's accounts. An overview of all the analytical stages is detailed as follows:

1. Pre-analysis stage: This stage consists of material selection (corpus) to be analyzed and its meticulous reading (Gondim and Bendassolli, 2014). Three authors (JC, GT, KJ) familiarized themselves with the interviews through reading and re-reading, with two members allocated to each transcript.
2. Encoding: Stage two involves defining and coding the content. During this stage, it is important to be clear about what is being coded and the rules for enumeration (presence or absence) (Gondim and Bendassolli, 2014). The sources of codes can be predefined (e.g., theory driven) or empirically created from reading and re-reading. For this study, an inductive empirical-based method was undertaken with each of the authors extracting data from each narrative into a proforma that included summaries (and corresponding quotes) of all the key factors (i.e., individual, structural, social) that were described as having a major impact on women's breastfeeding journey. Each narrative was checked against the transcript by a second reviewer who added any other factors thought to be important. Any differences were discussed and agreed by consensus.
3. Categorization: Stage three involves the organizational phase where the material is sorted into key criteria (Gondim and Bendassolli, 2014). During this stage all the authors reviewed the extracted data and produced a checklist of all the key factors detailed. This checklist was then used to record whether each factor was present (or not) within each women's account, and to produce descriptive (*n*, %) summary data. The factors were then grouped into system level themes, e.g., individual systems, dyadic systems, cultural/social systems, and health care systems.
4. Interpretation: This is the inferential process where interpretations are made of the data set (Gondim and Bendassolli, 2014); to look at the interrelationships between the codes and to begin model building (Iborra, 2007). At this stage, we decided to analyse the descriptive data based on whether women were or were not breastfeeding at 8 weeks. This enabled us to create two typologies of women who were breastfeeding (exclusively or mixed) or not breastfeeding, to assess and compare for variations in the system level factors and to help explain infant feeding outcomes. No statistical testing was undertaken so comparisons are drawn from the descriptive data only. Finally, one narrative from each typology that exemplified all the key factors was identified and case studies were produced (drawing on extracted quote material detailed within the pre-defined proformas).

### Ethics

Ethical approval for the ABA study was received from South-West—Cornwall and Plymouth Research Ethics Committee (16/SW/0336) on 28/11/2016. All participants were provided with a detailed information sheet that included information on why the study was being undertaken, how their data would be used and the voluntary nature of participation; all were asked to sign a consent form prior to data collection. Pseudonyms have been used for the case study descriptions.

### Reflexivity

We are a group of female researchers from psychology, midwifery, public health and health services research backgrounds. All of us were involved in the ABA feasibility study; two were involved in undertaking the interviews. All of us have breastfed our children to varying degrees and are all passionate about providing person-centered care that protects against poor mental being and promotes choice.

## Results

The included women were aged between 21 and 38 years, 17 (70.8%) were White British, half the sample were educated to degree level or higher, and all bar one were in paid employment (see Table 3). Overall, we found that while all of the 24 women had entered their breastfeeding journey with a strong intention to breastfeed, by 8 weeks post-natal nearly a third of them had discontinued breastfeeding. Categorical content analysis led to two typologies being identified within the data set, classified as “disappointed” (*n* = 7, 29.2%) and “by hook or by crook” (*n* = 17, 70.8%) (an English colloquial term describing using any means necessary to accomplish a goal). Women classified as “disappointed” were those who had stopped breastfeeding at the time of the 8-week questionnaire. All bar one (6/7, 85.7%) of the “disappointed” women, and 12/17 (70.6%) of “by hook or by crook” women had received the ABA intervention. An overview of the system level data for each typology is provided in Table 3.

**Table 3:** Different[-109mm] Q28 system level key factors by feeding typology.

	**Disappointed (*n* = 7)**	**By hook or by crook (*n* = 17)**	**Total**
*Individual level*			
Mother's age	21–32 years (mean 28 yrs)	22–38 years (mean 30.1 yrs)	
**Ethnicity**			
White British	6 (85.7%)	11 (64.7%)	17 (70.8%)
White other		4 (23.5%)	4 (16.7%)
Other/mixed	1 (14.3%)	2 (11.8%)	3 (12.5%)
**Education**			
GCSE	2 (28.6%)	1 (5.9%)	3 (12.5%)
A-level	2 (28.6%)	7 (41.2%)	9 (37.5%)
Degree/higher	3 (42.9%)	9 (52.9%)	12 (50.0%)
**Current work situation**			
Paid employment	6 (85.7%)	17 (100%)	23 (95.8%)
Unemployed	1 (14.3%)		1 (14.3%)
**What milk type do you want to give baby, first 6 months?**			
Breastmilk only	2 (28.6%)	10 (58.8%)	12 (50.0%)
Mainly breastmilk	3 (42.9%)	6 (35.5%)	9 (37.5%)
Half and half	2 (28.6%)	1 (5.9%)	3 (12.5%)
**Infant feeding intention**			
Committed breast feeder	3 (42/9%)	7 (41.2%)	10 (41.7%)
Probable breast feeder	4 (57.1%)	10 (58.8%)	14 (58.3%)
*Proactive help seeking*	4 (57.1%)	15 (88.2%)	19 (79.2%)
*Accessing wider support*	2 (28.6%)	17 (100%)	19 (79.2%)
*Socio-Cultural level system*			
**How were you fed as a baby?**			
Breastmilk entirely	3 (42.9%)	10 (58.8%)	13 (54.2%)
Formula milk entirely	1 (14.3%)	3 (17.6%)	4 (16.7%)
Breast and formula milk	3 (42.9%)	3 (17.6%)	6 (25.0%)
Don't know		1 (5.9%)	1 (4.2%)
Do you know anyone who has breastfed their baby?	7 (100%)	16 (94.1%)	23 (95.8%)
Positive experiences of support from personal networks	3 (42.9%)	16 (94.1%)	19 (79.2%)
Negative experiences of support from personal networks	3 (42.9%)	5 (29.4%)	8 (33.3%)
*Dyadic level systems*			
**Type of birth**			
Vaginal birth	2 (28.6%)	6 (35.3%)	8 (33.3%)
Planned C/S		2 (11.8%)	2 (11.8%)
Emergency C/S	1 (14.3%)	3 (17.6%)	4 (16.7%)
Assisted delivery	4 (57.1%)	6 (35.3%)	10 (41.7%)
Maternal complications intrapartum and/or post-natal (e.g., hemorrhage, transfusion)	3 (42.9%)	9 (52.9%)	12 (50.0%)
Infant complications post-birth (infant weight, NICU admission)	5 (71.4%)	2 (11.8%)	7 (29.2%)
Actual/perceived infant feeding complications (tongue tie, breastmilk insufficiency)	6 (85.7%)	10 (58.8%)	16 (66.7%)
*Health care level system*			
Unexpected top-ups of formula on post-natal wards	3 (42.9%)	2 (11.8%)	5 (20.8%)
Positive experience of hospital breastfeeding support	3 (42.9%)	13 (76.5%)	16 (66.7%)
Negative experience of hospital breastfeeding support	5 (71.4%)	8 (47.1%)	13 (54.2%)
Positive experience of community breastfeeding support	3 (42.9%)	13 (76.5%)	16 (66.7%)
Negative experience of community breastfeeding support	5 (71.4%)	4 (23.5%)	9 (37.5%)

Below we describe each of the system level themes and identify similarities and differences between the typologies by drawing on examples and quoted material. While important to note that numbers are small, particularly in the “disappointed” group, the analysis highlighted that whilst all the women started with positive antenatal intentions to breastfeed, there appears to be variations between the groups that help explain the differences in infant feeding outcomes. A summary table of the key variations is then provided, followed by a case study of each typology to demonstrate how these system level factors operate on a dynamic interacting basis.

### Individual Level System

These data concerned individual socio-demographic factors (e.g., age, ethnicity, education, employment status) and motivations for breastfeeding and help-seeking. Overall, women classified as “disappointed,” were younger and more likely to be White British compared to those in the “by hook or by crook” typology. In regard to motivation to breastfeed, while all the women included in this sample expressed strong intentions to breastfeed, less than a third of the women classified as “disappointed” planned to breastfeed exclusively, compared to over half of those classified as “by hook or by crook.” Some of the “disappointed” women were saddened by their decision to discontinue breastfeeding expressed through phrases such as “*I was gutted;”* others were more ambivalent; “*but it's better than having an unhappy and not healthy baby, so* [I] *accepted it*” (#12_ “disappointed”). Moreover, some still held positive beliefs around the importance of breastfeeding, and their willingness to “*try”* again with a future baby:

“*I am still a strong believer that breast is best, and as I say if I had my time again, I would try again*” (#2_ “disappointed”).

A further variation in motivation was also evident in terms of help-seeking behaviors. A higher percentage of women classified as “by hook or by crook” (88.2 vs. 57.1%) demonstrated proactive behaviors in seeking out support for breastfeeding (antenatally and/or post-natally). Furthermore, all the women within the “by hook or by crook” typology accessed wider community support vs. 28.6% of those classified as “disappointed.” This help-seeking behavior included attending antenatal classes, hypnobirthing, watching videos, undertaking research (reading books, internet), contacting professionals, and accessing breastfeeding groups:

“*I had a lot of help from midwives passing by—I was keen to ask for help as well*” (#3_ “by hook or by crook”).

For one woman in the “by hook or by cook” typology, this concerned contacting her ABA Infant Feeding Helper at points in which she “*felt like formula feeding”* (#5_ “by hook or by crook”). Whereas, for some of those classified as “disappointed,” they demonstrated a more passive approach of wishing the support had been proactively offered:

“*The midwives said, well if you need the help ask […] In hindsight, think it would have been helpful for someone to check you're doing it right*” (#2_ “disappointed”)

Some women spoke of difficulties in accessing local support due to, e.g., being unable to travel following an operative birth. Others held a misperception that available support was only targeted for those who were exclusively breastfeeding. One mother who was mixed feeding referred to knowing about the breastfeeding group but did not want to access it as “*I just felt like I wasn't a breast feeder anymore”* (#17_ “by hook or by crook”).

### Socio-Cultural Level System

This level concerns social and cultural-related factors on women's decision-making and behaviors. Similar percentages of women in both typologies had received breastmilk as a child; although on occasion, these experiences were limited. One woman reported “*my own mum mixed-fed me for 12 weeks”* (#21_ “disappointed”). All the “disappointed” women and all bar one of the “by hook or by crook” women knew someone who had breastfed. Women spoke of various ways in which these vicarious experiences affected their attitudes and behaviors, positively or negatively. One woman described how observations of a friend who was “*really focused on [breastfeeding] must, must, must do it”* had negative outcomes as “*she didn't get on very well with it at all*.” She therefore made a conscious decision to “*not to put too much pressure on myself to do it, I just tried and give it a go”* (#22_ “by hook or by crook”). Whereas for others, it was seeing the impact of not breastfeeding that positively influenced their decision:

“*My friend's baby has got quite ill quickly* [formula fed] *that's what made me decide”* (#8_ “by hook or by crook”).

From a counter perspective, some recounted stories of friends who were “*traumatized by breastfeeding not working out* […] *and who went on to formula feeding in the end”* (#25_ “disappointed”).

In regard to support from personal networks, while a higher percentage of women in the “by hook or by crook” typology received positive support (94.1 vs. 42.9%), similar percentages (~30–40%) of women in both groups had received negative support. Negative support included encouragement for formula feeding such as “*people bought me bottles as a gift even without asking what I planned to do”* (#9_ “disappointed”). Whereas for others, this related to active discouragement for breastfeeding; “*mum said “maybe you should quit* [breastfeeding]”” (#24_ “by hook or by crook”):

“*My partner was begging me to go onto the bottle. He was like “just go on the bottle I've had enough of talking about this””* (#25_ “disappointed”).

Positive support included recommendations for breastfeeding aids, such as an electric pump from a woman's sister-in-law; “*the electric pump saved it really, the breastmilk for him”* (#4_ “by hook or by crook”). There was also evidence, particularly expressed by women classified as “by hook or by crook” of partners and family/friends offering general household support: “*my husband off work until 5 weeks, made it a lot easier, could breastfeed lots”* (#15_ “by hook or by crook”); “*amazing friends* […] one *rocks up in her pajamas, cooked me loads of lasagnes”* (#20_”by hook or by crook”). Another described how her mother:

“[She was like] *no it's fine you can sit here for an hour it doesn't matter, I will cook dinner, I will do your washing, I will do your ironing, I'll hoover the house.”* (#30_ “by hook or by crook”).

Others referred to receiving ongoing encouragement by family; “*my partner was very supportive”* [of breastfeeding] (#27_ “by hook or by crook”), and friends; “*my friend said ‘just keep going with it'* [breastfeeding]*, she loves breastfeeding* (#9_ “disappointed”).

### Dyadic Level System

Dyadic systems related to mother-infant factors around the time of birth or post-natal period that could influence breastfeeding; whereby it is recognized that infant feeding takes place within a reciprocal responsive dyadic system. Overall, there were similar percentages of women who had experienced intervention(s) during birth (~60–70%) or had experienced complications during the pregnancy, intrapartum and/or post-natal period (~40–50%). These complications included gestational diabetes, post-partum hemorrhage, blood transfusions, “*bad fungal rash on groin and then breasts that meant I couldn't really walk”* (#20_ “by hook or by crook”), or an intervention-based and/or “*traumatic long birth”* that created delays in early skin to skin contact:

“*I had quite a bad experience that they overdosed me, so I weren't really in the frame of mind to put her straight on me*” (#10_ “disappointed”).

Some women reported experiencing no breastfeeding problems in the immediate post-natal period; “[baby went] *straight onto breast*” (#2_ “disappointed”): “*I didn't have any problems at all, she just latched on 2 h after being born”* (#16_ “by hook or by crook”). However, a key difference between the groups was that a higher percentage of babies of women classified as “disappointed” had experienced complications in the early post-natal period (71.4 vs. 11.8%), and “disappointed” women were more likely to have considered their babies to have experienced actual or perceived infant feeding complications (85.7 vs. 58.8%). Complications related to issues such as fetal distress following an intervention-based birth, obstetric cholestasis in the liver, and admissions to the neonatal unit, which for one baby was due to a 20% drop in birth weight, dehydration, and jaundice. Some of the “disappointed” women considered the birth to have been directly responsible for feeding difficulties. For example, one woman reported how her son had been “*really pulled about”* during a ventouse delivery, which led to “*back, neck, mouth, and jaw problems, making feeding difficult”* (#25_ “disappointed”).

Other complications associated with infant feeding that emerged over the early post-natal period included weight loss, tongue tie, reflux, mastitis, the level of nipple pain that women experienced, or concerns of breastmilk insufficiency; [baby was] “*not getting enough”* (#10_ “disappointed”):

“*When we went to get discharged the health professionals had concerns about weight loss and I did not take it very well, I burst into tears”* (#20_ “by hook or by crook”).

### Health Care Level System

Factors operating at this level concern the quality and types of support women received from professionals, including those working within the hospital, community and *via* the ABA Infant Feeding Helpers. Overall, a higher percentage of babies of women classified as “disappointed” had received an unexpected top-up of formula milk during their hospital stay (42.9 vs. 11.8%). Women often did not describe this in negative terms due to it being perceived as a necessity as, e.g., “[the midwives told me] *he's not feeding, so we're going to top him up with a bit of formula*” (#20_ “by hook or by crook”). Although one referred to how the introduction of a top-up made her decision to “*solely formula feed”* as she “*just wanted to get something into her, wanted to get her better”* (#2_ “disappointed”).

A higher percentage of women classified as “by hook or by crook” had received positive support from professionals both during the hospital stay, and in the community (76.5 vs. 42.9% respectively). Positive support included offers of reassurance, encouraging women to use available support, “*she* [ABA Infant Feeding Helper] *kept telling me about breastfeeding groups*” (#25_ “disappointed”), and the receipt of individualized responsive care. One woman who had had a cesarean reported:

“*It's very difficult in the early days to lift and stuff like that, so yeah it was then a bit challenging back on the ward, but every single staff member was so amazing at helping me get the position for him right, and he just took to it”* (#23_ “by hook or by crook”).

Others referred to how treatment and/or advice had helped to resolve infant feeding issues, such as being prescribed antibiotics which helped to resolve her mastitis, “*advice on how to feed without nipple shields”* (#15_ “by hook or by crook”), or lanolin for nipple pain. On occasion, positive support concerned “permission” for combination feeding which in term encouraged women to continue breastfeeding:

“*The fact that combination feeding was allowed, because that meant we knew he was getting something and it allowed me to continue”* (#4, “by hook or by crook”).

“Disappointed” women were more likely to receive negative support from healthcare staff, in the hospital (71.4% vs. 47.1%), and within the community (71.4% vs. 23.5%). Accounts of negative support included ineffective, “*strict”* and/or “*rushed”* support often associated with health professionals not prioritizing support for breastfeeding”[healthcare professionals] *had a lot of more important things to do”* (#24_ “by hook or by crook”) or being “*too busy”* to offer support:

“*Maybe if I'd have said to the midwives ‘look can you show me one more time what to do' but they're so busy in there”* (#21_ “disappointed”).

Some women also referred to requests for support not being followed up; “*I explained the situation to her* [ABA Infant Feeding Helper]*, and that's all that happened really”* (#9_ “disappointed”); or support being provided at a time when women had limited capacity:

“*Midwife advised on different positions, tried to give lots of help but* [I was] *too tired to take it in*” (#15_ “by hook or by crook”)

Other complaints concerned conflicting advice, and leaving hospital without breastfeeding having been established:

“*I ended up giving him a bottle in the hospital and discussed with the midwives about me continuing to hand express at home. They advised that I shouldn't really do that, and I was that desperate to get home at that point I just said, ‘okay well, I'll carry on trying at home' I wasn't getting any help in the hospital, so I said I'm not gaining anything staying*” (#9_ “disappointed”).

Below we detail a summary (Table 4) of the key variations between the groups and present two case studies, one from each typology to demonstrate the interaction of multiple system-level factors interacting with women's motivation and capacities to breastfeed.

**Table 4:** Summary profiles of the two typologies: “Disappointed” and “By book or by crook.”

**Disappointed**	**By hook or by Crook**
More likely to (be/have): • Younger • White British • Plan to mixed feed • Infant to have complications post-birth • Perceive baby to have infant feeding complications • Baby more likely to receive unexpected top-up of formula milk in hospital • Negative experiences of breastfeeding support in hospital • Negative experiences of support from personal networks • Negative experiences of community breastfeeding support	More likely to (be/have): • Older • Plan to breastfeed exclusively • Proactive at help seeking • Accessed wider support • Positive experiences of breastfeeding support in hospital • Positive experiences of support from personal networks • Positive experiences of community breastfeeding support

#### Gazala (by Hook or by Crook)

Gazala is 28 years old and as she is not originally from the UK, she has no family support locally available; “*I have sisters and I always can ask them. But it's another country, it's not my own country also you don't know so many people*.” Gazala had gestational diabetes so expressed colostrum before the birth and froze the milk. She took this milk to hospital with her. She had a cesarean section which was very quick, but painful afterwards, making movement hard. Gazala's baby latched on just after he was born. During the first night “*my husband was with me which was good*,” but the second night he couldn't stay because the “*hospital doesn't allow you to, that night was really difficult. So it didn't help really with the breastfeeding*.” Gazala felt that the midwife didn't have time to support her and then when the midwife came and latched baby on Gazala thought, “*what am I go to do now?*” Overall, Gazala feels that she didn't get enough help at the hospital. She went home after the second night due to there being insufficient support (and noise/disruption of others). An issue with tongue-tie was identified in hospital and this was fixed before discharge. Breastfeeding continued to be very painful. Gazala's husband and own mother were upset. Gazala's mother visited from before birth until baby was 6 weeks old—they tried, creams, nipple shields. Thinking she couldn't continue, Gazala's husband said it was okay just to have tried and was supportive of whatever she did. Her husband went out to buy formula—but the baby was asleep by the time he got back. Gazala also found it helped to express and feed her baby expressed breast milk to rest her nipples. Gazala found her community midwife and health visitor to be helpful, they checked the baby's latch, and one of them (she couldn't remember which) recommended the breastfeeding counselor at the breastfeeding group. Unfortunately, because of having had a cesarean, Gazala couldn't get to the breastfeeding group until her baby was 6 weeks old. She did start attending after 6 weeks and found that it helped a lot that the local breastfeeding counselor attended the group. Gazala's ABA Infant Feeding Helper also provided reassurance and information. Gazala also talked to her health visitor and GP. The breastfeeding counselor called to see how she was getting on and Gazala has attended the breastfeeding group a few times, as well as other groups at the Children's Center. After 2 months the pain associated with breastfeeding finally subsided. At the time of the interview, Gazala's baby had ~7 bottles of formula, partly in preparation for Gazala's return to work.

#### Catherine (Disappointed)

Catherine is White British and aged 21 years. She says that before she had her baby, “*my biggest anxiety was breastfeeding*.” She had a friend who had been traumatized by breastfeeding not working out. Another friend tried everything and went on to formula feeding in the end. “*So I had seen these people around me really struggle*.” For these reasons, while she “*really wanted”* to breastfeed, she felt that mixed feeding was more likely. Catherine's baby was born following an induction and ventouse delivery. While her baby latched on “*straight away no problem*,” she felt the method of delivery led to problems with back, neck, mouth, and jaw, making feeding difficult. Catherine's experience of hospital staff was that they were too busy to support women. Her baby was also given a top-up, and Catherine considered this a positive decision as at least he was “*getting something.”* She felt confident to go home the day after giving birth as feeding “*wasn't too bad in hospital*,” although in hindsight wishes she had stayed for longer. At home, she found breastfeeding to be a “*complete pain*”, “*he couldn't do it*.” A midwife came out, but Catherine says, “*I think for a lot of them it was a bit overwhelming … because I was just too emotional. I struggled with anxiety in my life before… so I think they were a little bit worried about me*.” While the health professionals provided advice, she felt they did not give her the practical support that she needed. While the ABA Infant Feeding Helper was seen as supportive and encouraged her to access wider support, Catherine said, “*I shut the outside world out really, which I know is silly, I should have looked for help*.” Her mother-in-law had planned to be around to help Catherine after the birth, but was looking after a sick relative, so was unable to help. Catherine's infant continued to lose weight, and the health visitor came “*quite often.”* She eventually visited a cranial osteopath—when she “*realized what the problem was*.” In the end, she says, “*My partner was begging me to go onto the bottle.”* Catherine found her baby to be happier on formula and started to gain weight. Looking to the future, Catherine says she would try breastfeeding again, “*because of the guilt, I would want to do it again.” I would be like “I have to do it this time.”*

## Discussion

In this paper we present a secondary analysis of qualitative data that uses complex systems thinking to understand how different factors operating at different levels interact and influence women's motivations and experiences of breastfeeding. Our findings highlight the importance of individual factors, family and social networks and health professional support on women's infant feeding journeys, and supports the model in which infant feeding needs are perceived within a socio-ecological system of factors (Trickey, 2018). A 2016 Lancet series on breastfeeding and public health present a strong argument for the need for an ecological approach to infant feeding, arguing that increases in breastfeeding rates and consequent health gains were unlikely to be achieved while public health attention remains focused on educating pregnant women about the benefits of breastfeeding (Rollins et al., 2016). Furthermore, a review of reviews that investigated the evidence for breastfeeding interventions against the social-ecological model concludes that while a multilevel systems level approach is crucial, to date most interventions focus on health service settings, with insufficient attention paid to policy and structural interventions and the workplace (Tomori et al., 2022).

We identified two typologies of women who had planned to breastfeed: “disappointed” and “by hook or by crook.” While the number of women, particularly within the “disappointed” group were small, different influences on their infant feeding journeys were identified. Those who had a breastfeeding experience that we characterized as “disappointed” displayed a strong motivation to breastfeed antenatally but were also prepared for failure (through more women considering mixed feeding) and had stories from other women about the difficulties that they had faced. These stories which are often fatalistic and describe disappointing experiences are an important part of the context for the mothers coming up behind. The term “disappointed” has been previously used to depict those who were unable to meet their breastfeeding intentions (Trickey, 2018), and the guilt and shame experienced by women who end up switching to formula feeding before they planned has been reported previously (Thomson et al., 2015; Jackson et al., 2021). Infants of disappointed mothers were more likely to receive an unexpected top-up on the post-natal ward, which is known to undermine maternal confidence and milk production (Royal College of Paediatrics Child Health, 2017). “Disappointed” mothers were also more likely to experience infant feeding difficulties, such as perceived breastmilk insufficiency. While on one hand this may be associated with top-ups, perceptions of failure such as a self-fulfilling prophecy, or wider psychological factors (Huang et al., 2022; Segura-Pérez et al., 2022), infants of “disappointed” mothers were more likely to experience complications, such as jaundice and neonatal admissions; with the link between complications and a higher risk of non-exclusive breastfeeding being reported by others (Gianni et al., 2019; Segura-Pérez et al., 2022). Our findings align with those from a recent review that found relationships between lactation problems and perceived breastmilk insufficiency, maternal confidence, breastfeeding duration and discontinuation, and use of formula milk (Vilar-Compte et al., 2022b).

“Disappointed” women were also more likely to experience negative support from healthcare professionals, both within the hospital and the community. The detrimental impact of inappropriate and conflicting information and support from healthcare staff is well-reported (Schmied et al., 2009; Thomson and Dykes, 2011; Johnson et al., 2016; Tomori, 2022). While healthcare professionals may lack the confidence, knowledge, and skills to provide breastfeeding support (Yang et al., 2018), others argue that health professionals are influenced by the formula industry (Van Tulleken, 2018). While this emphasizes the need for staff to receive appropriate training that is free of commercial influence, it also calls for further research that investigates the unconscious and conscious biases associated with the formula industry amongst healthcare staff. “Disappointed” women expressed a need for more proactive support for breastfeeding and displayed limited help-seeking behaviors. Identifying women who are already somewhat resigned to the possibility of failure antenatally, and whose infant has experienced complications might enable additional targeted proactive support to be offered. Breastfeeding support delivered proactively was identified as key to successful peer support in a realist review (Trickey et al., 2018) and shown promising results when delivered in feasibility trials (Hoddinott et al., 2012; Clarke et al., 2020a).

In contrast, women we describe as “by hook or by crook” reported a high level of motivation to exclusively breastfeed in the antenatal period, they were more likely to report positive support from family and friends and health care professionals and displayed help-seeking behavior to address their informational and practical needs to enable them to successfully breastfeed. Some authors (Brown et al., 2011; Thomson and Dykes, 2011) have identified that determination, or “meaningfulness” associated with breastfeeding is an important characteristic of breastfeeding continuation, with these women breastfeeding despite experiencing difficulties. Motivated women often develop strategies to overcome detrimental and/or ineffective support (such as finding help in the community or through family and friend networks or privately) (Thomson and Dykes, 2011). A strategy to help facilitate these behaviors is through assets-based approaches, ensuring information is readily available and through peer support interventions. The ABA feasibility study and ongoing ABA-feed trial explicitly encourage women to access information and support through their personal, social and community networks (Clarke et al., 2020a; Ingram et al., 2020; Thomson et al., 2020).

Our findings highlight that to improve breastfeeding rates, interventions need to tackle more than one level of the socio-ecological system, for example by providing information and support for women (Thomson and Dykes, 2011), supporting them to develop self-efficacy for breastfeeding (Blyth et al., 2002; Bartle and Harvey, 2017) addressing the needs of partners (Brown and Davies, 2014; Sihota et al., 2019), family (Ingram et al., 2003), and delivering community support (Thomson and Dykes, 2011; Thomson et al., 2012). In addition, health services need to address the barriers to receiving adequate support perceived by women, such as the sense that health professionals are too busy (Schmied et al., 2009) and a lack of sufficient access to midwifery support in the early post-natal period (Plotkin, 2017). Most participants in our study did not reflect on challenges about breastfeeding when returning to work, likely due to the timing of the interviews. However, insights from one of the case studies (Gazala) indicates similar issues to others (Desmond and Meaney, 2016; Snyder et al., 2021) in terms of how employment can influence women's infant feeding decisions. The need for specific interventions and legislation to ensure workplace breastfeeding protection is highlighted (Snyder et al., 2021; Tomori et al., 2022).

Our study has multiple strengths. The study provides an in-depth qualitative examination of women's experiences of infant feeding support. With our links to the trial data, we were able to identify the infant feeding intentions of women prior to giving birth, so avoided any *post-hoc* rationalizations in response to lived experience of feeding; this is a limitation of many qualitative studies with data collected in the post-natal period only (Schmied et al., 2009). Our data analysis was undertaken independently in duplicate and then inconsistencies discussed and agreed. The consistency of many of our findings with previous research findings validates our results. The women who took part were broadly representative of the UK population in terms of age of first birth and ethnicity, though the number of women from non-White backgrounds was very low. The limitations are that the sample size is small, particularly in regard to “disappointed” women. It is also likely that there are further typologies such as women who face no real complications, or those with more ambivalent experiences, with further testing of the variations in women's journeys needed. A larger sample size would also allow more detailed investigation into the social inequities that may be involved in who decides to breastfeed in the first place and who can persist in breastfeeding over time. The participants were taking part in a feasibility trial of an infant feeding support intervention and agreed to a qualitative interview, so may not be fully representative of all women with an intention to breastfeed. However, the intervention was purposively framed as infant feeding, not breastfeeding support and was inclusive of women regardless of their feeding intention. The interviews were also undertaken by researchers from the ABA feasibility study and the participants will have been aware of their links to the study which may have influenced their responses. Notwithstanding these issues, to our knowledge this is the first study to use a complex systems lens on qualitative data to understand how different system level factors interact to influence women's breastfeeding journeys. While further research is needed, it emphasizes, as reported by others (Tomori et al., 2022), that structural changes and multi-level interventions are needed to enable women to fulfill their breastfeeding intentions.

## Data Availability Statement

The original contributions presented in the study are included in the article/supplementary material, further inquiries can be directed to the corresponding author.

## Ethics Statement

The studies involving human participants were reviewed and approved by South-West—Cornwall and Plymouth Research Ethics Committee (16/SW/0336) on 28/11/2016. The patients/participants provided their written informed consent to participate in this study.

## Author Contributions

GT, KJ, JI, and JC made substantial contributions to the conception of the work, were involved in reading the narratives, and devising and applying the data extraction tool. JC and DJ were involved in data acquisition. GT led on the interpretation of the data, with all final interpretations agreed by GT, JI, JC, DJ, and KJ. GT drafted the full manuscript, with all authors contributing to separate sections. All authors reviewed the content, provided approval for publication, and agreed to be accountable for all aspects of the work. All authors contributed to the article and approved the submitted version.

## Funding

This paper presents independent research commissioned by the National Institute for Health Research (NIHR). The funders had no involvement in the design of the study, the collection, analysis, and interpretation of data and in writing the manuscript. The views and opinions expressed by authors in this publication are those of the authors and do not necessarily reflect those of the NHS, the NIHR, NETSCC, the Public Health Research programme or the Department of Health and Social Care. This project was funded by the Public Health Research programme (15/53/04). KJ is part-funded by NIHR Applied Research Collaboration - West Midlands.

## Author Disclaimer

The funders had no involvement in the design of the study, the collection, analysis, and interpretation of data and in writing the manuscript. The views and opinions expressed by authors in this publication are those of the authors and do not necessarily reflect those of the NHS, the NIHR, NETSCC, the Public Health Research programme, or the Department of Health.

## Conflict of Interest

KJ is part-funded by NIHR Applied Research Collaboration - West Midlands. The remaining authors declare that the research was conducted in the absence of any commercial or financial relationships that could be construed as a potential conflict of interest.

## Publisher's Note

All claims expressed in this article are solely those of the authors and do not necessarily represent those of their affiliated organizations, or those of the publisher, the editors and the reviewers. Any product that may be evaluated in this article, or claim that may be made by its manufacturer, is not guaranteed or endorsed by the publisher.
